# Environmental and occupational respiratory diseases – 1049. Impacts of air pollution and enviornmental tobacco smoking on the symptoms of childhood asthma

**DOI:** 10.1186/1939-4551-6-S1-P48

**Published:** 2013-04-23

**Authors:** Chang Sun Sim, In-Bo Oh, Cheol-in Yoo, Yangho Kim, Ji-Ho Lee

**Affiliations:** 1 Occupational and Environmental Medicine, Ulsan University Hospital, College of Medicine, University of Ulsan, Ulsan, South Korea; 2 Environmental Health Center, College of Medicine, University of Ulsan, South Korea

## Background

Childhood asthma is one of the most common public health problems in developed communities. It is associated with many environmental factors such as air pollutant exposure, parental smoking, climate condition, and aeroallergens. The aim of this study was to examine the combined effects of environmental tobacco smoking and air pollution on the symptoms of childhood asthma.

## Methods

A cross-sectional survey of asthma and allergic diseases in elementary school children was conducted in July 2009. The survey was carried out for all six graders (aged 5-14 years) via parental questionnaires (including the ISAAC core questions) at randomly selected two schools (A and B) which are located in suburban residential area and near industrial complexes, respectively. Data on the lifetime and 1-year asthma symptom prevalence and environmental factors were collected from 2,201 children (School A: n=882, School B: n=1,319) participated in the study. All data were analyzed with SPSS 18.0 and odds ratio was calculated by logistic regression analysis to estimate the combined effects of study factors on Asthma.

## Results

Survey results showed that the prevalence rates of asthma symptoms (lifetime/1-year symptoms) were higher for school B (13.8/4.5%) than the rates for school A (9.6/2.8%). The prevalence rates are also relatively higher by 1~2% for a group exposed to environmental smoking (ETS) than the other group. In multivariate logistic analysis, it was found that the significant risk factors for lifetime and 1-year symptom of asthma were maternal history of allergy, age (<10 years), air pollution (AP) (+), ETS(+). The combined effect between AP and ETS were significantly associated with lifetime and 1-year symptoms of childhood asthma.

The multivariate model indicated that adjusted odd ratios of lifetime and 1-year symptoms of asthma were 1.682 (95% CI: 1.092-2.592) and 2.276 (95% CI: 1.117-4.638), respectively, between the groups with both AP(+) and ETS(+) and the others.

## Conclusions

Our results suggest that living in polluted area and exposure to tobacco smoking are significantly associated with the prevalence of symptoms of childhood asthma.

